# Modification of Collagen Film via Surface Grafting of Taurine Molecular to Promote Corneal Nerve Repair and Epithelization Process

**DOI:** 10.3390/jfb13030098

**Published:** 2022-07-17

**Authors:** Yang Liu, Chuanlei Zhang, Yanhui Kong, Huiyu Liu, Jia Guo, Hui Yang, Linhong Deng

**Affiliations:** 1 Institute of Biomedical Engineering and Health Sciences, Changzhou University, Changzhou 213164, China; zhangcl@cczu.edu.cn (C.Z.); kongyh@cczu.edu.cn (Y.K.); liuhy@cczu.edu.cn (H.L.); guojia@cczu.edu.cn (J.G.); 2 Key Laboratory of Prevention and Treatment of Cardiovascular and Cerebrovascular Diseases, Ministry of Education, Gannan Medical University, Ganzhou 341000, China

**Keywords:** collagen, taurine, nerve repair, epithelization, artificial cornea

## Abstract

Corneal defects can seriously affect human vision, and keratoplasty is the most widely accepted therapy method for visual rehabilitation. Currently, effective treatment for clinical patients has been restricted due to a serious shortage of donated cornea tissue and high-quality artificial repair materials. As the predominant component of cornea tissue, collagen-based materials have promising applications for corneal repair. However, the corneal nerve repair and epithelization process after corneal transplantation must be improved. This research proposes a new collagen-based scaffold with good biocompatibility and biological functionality enhanced by surface chemical grafting of natural taurine molecular. The chemical composition of collagen-taurine (Col-Tau) material is evaluated by Fourier transform infrared spectroscopy and X-ray photoelectron spectroscopy, and its hydrophilic properties, light transmittance, swelling performance and mechanical tensile properties have been measured. The research results indicate that the Col-Tau sample has high transmittance and good mechanical properties, and exhibits excellent capacity to promote corneal nerve cell growth and the epithelization process of corneal epithelial cells. This novel Col-Tau material, which can be easily prepared at a low cost, should have significant application potential for the treating corneal disease in the future.

## 1. Introduction

The human cornea is the transparent organ that is crucial for normal vision. Blindness due to damage and disease from mechanical, thermal, microbial infections and chemical injuries frequently lead to an impaired dysfunction of the cornea [1]. Currently, keratoplasty is considered the clinic gold standard for therapy of corneal blindness [2,3]. However, the transplantation of allogeneic tissue is a useful method due to the good accessibility and low immunity of the native cornea. But, the availability of high-quality donor corneal tissue is inadequate worldwide [4,5]. In addition, some donated corneas may stimulate immune rejection and lead to a risk of infection after implantation [6]. To mitigate this problem, many researchers have tried to prepare various artificial materials using different macromolecules to reconstruct cornea scaffolds that mimic normal corneal tissue [7,8,9,10]. Collagen fibrils, as the predominant component of the human cornea, with good biocompatibility, biodegradability and biological activity, have been extensively studied for corneal repair and regeneration [11,12,13].

An ideal artificial corneal scaffold should possess several critical physical features for clinical utility, including appropriate water-uptake capability and permeability, high light transmittance, excellent mechanical strength, biocompatibility, etc. [14,15]. In many previous studies, although different kinds of collagen-based films had been suggested to replace the pathologic cornea in various ways, corneal nerve repair and epithelization process is difficult to achieve quickly after the implantation is still the problem of many artificial corneas [16,17]. Recently, researchers attempted to promote corneal epithelialization by introducing growth factors into the scaffolds or by improving the surface structure of the artificial corneal material [18,19]. Although these methods can promote the adhesion process and the proliferation rate of human cornea cells on the films, they can still have limitations. The optical transparency of these films often exhibits a certain reduction as a result. For this reason, methods that introduce natural macromolecules with specific bio-functions on the surface of artificial corneal have been developed in corneal tissue engineering [20,21]. 

Taurine is a kind of sulfur-containing amino acids commonly isolated from ox [22,23]. Several scientific studies have reported on the cell-protective properties of taurine molecular, which include anti-oxidation, maintenance of cell morphology, anti-apoptosis, neurotransmission, and osmoregulation [24]. Moreover, it’s worth noting that taurine can improve visual function and reduce the risk of cataracts at the same time [25]. Indeed, taurine is one of the most abundant amino acids in several tissues of the human eye, such as ciliary bodies, iris and cornea [26,27]. Besides, the unique structure and chemical functional groups of taurine molecular allow it combines with collagen molecular by chemical crosslinking [28]. Although taurine is present in human eyes, few results reporting the physiological functional role of taurine in the various eye structures are available. Considering the above characteristics of taurine, the role of this molecular in promoting nerve growth may be demonstrated in the functional design of corneal repair materials. 

In this research, taurine molecular was bonded to the smooth surface of collagen membrane with high optical transparency by using 1-Ethyl-3-(3-dimethylaminopropyl) carbodiimide (EDC) and N-hydroxy succinimide (NHS) as the crosslinking agent and catalyst, respectively. The chemical composition and element content of collagen-taurine (Col-Tau) film were tested by XPS and FTIR, and its physicochemical properties and biological performance were also evaluated. The biocompatibility of Col and Col-Tau samples to human cornea epithelial cells (HCECs) and PC12 cells was also observed. The purpose of the research is to investigate the viability of adopting chemical grafting of taurine molecular on the surface of collagen film to promote corneal nerve growth and the epithelization process. 

## 2. Materials and Methods

### 2.1. Materials

Taurine and paraformaldehyde were purchased from Sigma Chemical Company, USA. Type I collagen was supplied by HM Biotech Ltd., China. Phosphate buffered saline (PBS, pH = 7.4) was prepared from tablet form (Calbio Chem Corp, Germany). GL Biochem Ltd. (Shanghai, China) supplied 1-Ethyl-3-(3-dimethyl aminopropyl) carbodiimide (EDC) and N-hydroxy succinimide (NHS). All of the cell-culture related reagents used in this research were supplied by Sigma Chemical (St. Louis, MO, USA). The deionized water was obtained from a water purification system (Millipore S.A.S., Guyancourt, France). The PC12 neuronal cell line in this research was provided by the Type Culture Collection of the Chinese Academy of Sciences, Shanghai, China. Human corneal epithelial cells (HCECs) in this research were provided by the School of Ophthalmology and Optometry and Eye Hospital, Wenzhou Medical University, China. The protocol of the cell experiments was approved by the Ethics Committee of Changzhou University. All other chemical reagents were of analytical grade.

### 2.2. Preparation Method of Collagen-Taurine Material 

**Scheme 1:**
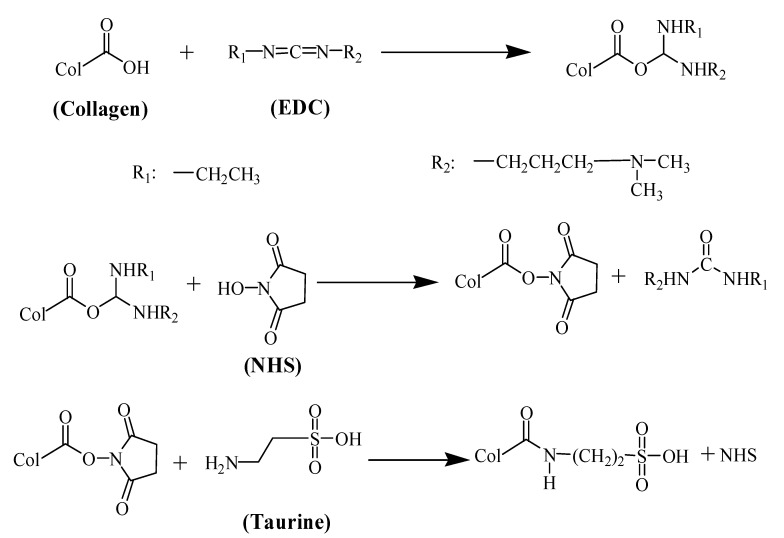
Proposed mechanism diagram of crosslinking reaction between collagen molecular and taurine.

For the collagen sponge and taurine powder, the collagen sponge was dissolved in 0.01 mol L^−1^ HCl and taurine was dissolved in distilled water with a concentration of 10 mg mL^−1^. Then the collagen solution (about 8.5 mg mL^−1^) was air-dried to form a cornea-shape film under sterile conditions. The air-dried collagen film was immersed into 10 mL taurine solution (10 mg mL^−1^). Then, EDC and NHS were added to crosslink the collagen and taurine (the mass ratio of EDC:NHS:Col-Tau is 1:1:6). The chemical cross-linking process was conducted by stirring the mixture solution slowly for 6 h. Afterward, the obtained Col-Tau film was dried and rinsed three times with distilled water. For comparison purposes, Col samples without taurine modification are also fabricated as blank group. The Scheme 1 indicates the chemical schematic illustration of crosslinking reaction routes between taurine molecular and collagen. 

### 2.3. XPS of Col and Col-Tau Film

The changes in the chemical composition of the film’s surface and element content of the two collagen-based films were evaluated by the XPS test. A Kratos Analytical (UK) model Axis Ultra system with an Al K_α_ X-ray source (*hν* = 1486.6 eV, 150 W) was used in the XPS spectrometer and the C1s of C–C (about 284.6 eV) was chosen as the reference line. Besides, the wide scanning was operated with pass energy of 140 eV, while the high-resolution region scans were gathered with the pass energy of 55 eV. In addition, analysis of the surface of different samples and quantification results of the two samples were obtained. 

### 2.4. FTIR of Col and Col-Tau film

The FTIR spectrum of the Col and Col-Tau sample was evaluated by a Fourier Transform Infrared-Attenuated Total Reflectance spectrometer (FTIR-ATR, Vector 33, Bruker, Germany). The spectra were obtained within the wave number range of 4000–350 cm^−1^. 

### 2.5. Contact Angle (CA) on the Surface of Col and Col-Tau

The contact angle tests were carried out on Data Physics OCA 15 (Germany). An amount of 1 µL of water droplets was dropped onto the surface of two collagen-based films with a syringe. Then, the static contact angle was evaluated at various subsequent time points. Every result indicated in the study was an average value of 5 tests.

### 2.6. Swelling Performance after Water Absorption of Col and Col-Tau

The water saturation of the two samples was determined in PBS. The equilibrated water content of the two kinds of the collagen-based sample was measured according to the following method. Every result was an average value of 10 tests. The saturated water absorption of the samples was calculated according to the following formula:

Absorption of water = (W_t_ − W_0_)/W_t_ × 100%; (1)

Here, W_t_ is the final wet weight of the collagen-based films, and W_0_ is the initial dry weight of the samples.

Before testing, two kinds of collagen-based sample with known sizes and thicknesses were swelled in PBS. The wet sizes of the Col and Col-Tau were measured at target times. Variations in thickness and surface area were calculated according to the formulas:

Increase of thickness = H_t_/H_0_; (2)

Increase of surface area = S_t_/S_0_; (3)

Here, H_t_ and S_t_ indicate the final sizes of the wet samples, respectively, while H_0_ and S_0_ indicate the initial sizes of the dry films, respectively.

### 2.7. Optical Properties Test of Col and Col-Tau Film

Before measuring the light transmission of the collagen films, the samples were immersed in Phosphate Buffered Saline. The thickness of the pre-wetted Col-Tau sample is about 230 ± 25 μm. Then, the wet collagen films were fixed on the ultraviolet–visible spectrophotometer to evaluate the optical properties (UV3802, Synergy, Bio-Tek, CA, USA). 

### 2.8. Mechanical Performance of Col and Col-Tau Film

Tensile testing was performed on the two kinds of collagen-based samples using a uniaxial loading device (Instron Corporation, Model #5567, WA, USA). Before the tensile test, the Col and Col-Tau films were immersed in PBS for saturated water absorption.

### 2.9. Proliferation of PC12 Neuronal Cells on Col and Col-Tau Film

The PC12 neuronal cell line is used as a model system to study nerve growth [29,30]. To measure the growth rate of the PC12 cells. PC12 cells were maintained routinely in RPMI 1640 medium, which was supplemented with 5% fetal calf serum and 10% heat-inactivated horse serum plus 100 μg/mL streptomycin and 100 units/mL penicillin at 37 °C. The PC12 cells were treated with bulbocapnine (5–40 μM) and then incubated in an incubator for one day. After the two collagen-based materials were transferred to the bottom of the Petri dishes, the PC12 cells were inoculated to the surface of Col-Tau film and Col film. The metabolic activity of the PC12 cells was evaluated by MTT assay.

### 2.10. HCECs Growth on the Surface of Col and Col-Tau

In this study, HCECs were provided by Ophthalmology and Optometry and Eye Hospital in Wenzhou, China. The cells were incubated under 5% CO_2_ at 37 °C, and cultured in DMEM (Waltham, MA, USA) with high glucose, supplemented with 15% fetal bovine serum (Gibco BRL), 5 μg mL^−1^ insulin, 100 U mL^−1^ penicillin, 5 μg mL^−1^ human transferrin (Sigma-Aldrich, MA, USA), 2 mM L-glutamine, 100 μg mL^−1^ streptomycin (HyClone) and 10 ng mL^−1^ human epidermal growth factor (EGF; Gibco BRL). The Col-Tau sample was washed three times in PBS and sterilized by UV radiation for about 2 h before cell experiments. Then, the pre-wetted sterile sample was used for cell culture and the seeded density of HCECs was 5000 cells/cm^2^. During cell culture, the cell culture medium was changed every two days. The change of HCECs on the surface of film was observed under an inverted phase contrast microscope (Zeiss Observer A1). Moreover, the metabolic activity of the HCECs was determined by MTT assay.

### 2.11. Histology

After the corneal epithelial cells were cultured with Col-Tau films for about seven days, the Col-Tau films were lightly washed once with PBS and then fixed with 4% paraformaldehyde at room temperature for 24 h. Then, the wet films were dehydrated using ethanol, subsequently immersed in xylene and finally embedded in paraffin. For ECM identification, the sections of the sample were stained with standard hematoxylin and eosin (H&E staining,). Three replicates were used for each sample.

### 2.12. Statistical Analysis

All test results are shown as the mean ± standard deviation. The statistical analysis was performed using analysis of variance (ANOVA) to analyze the significant differences among the groups. A level of *p* < 0.05 was considered to be statistically significant.

## 3. Results and Discussion

### 3.1. X-ray Photoelectron Spectroscopy

**Figure 1:**
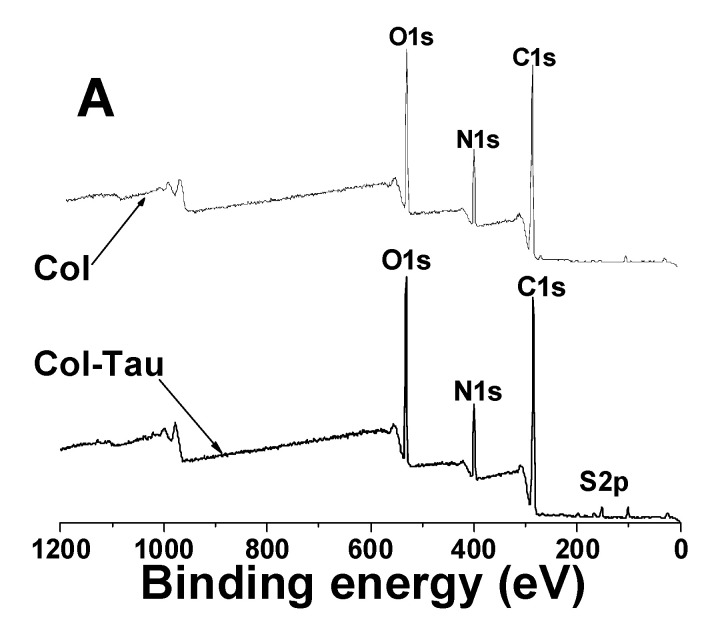

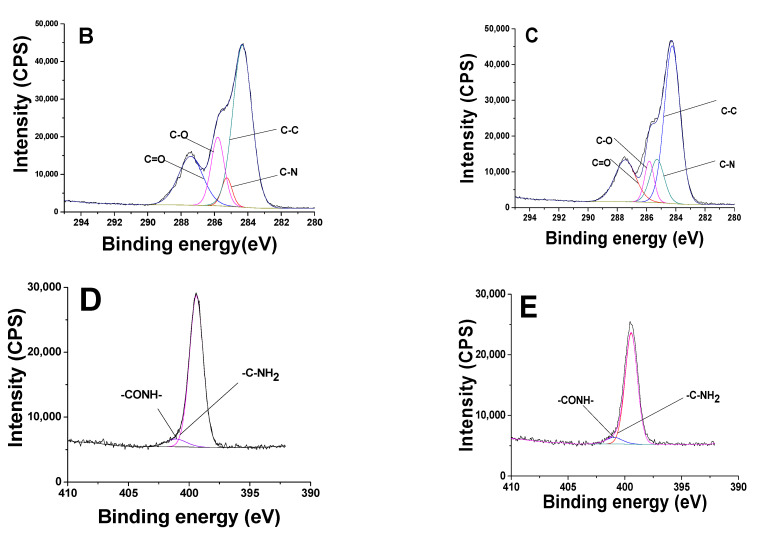
XPS spectra indicate the chemical composition on the surface of Col and Col-Tau. After chemical modification by the taurine, sulfur element appears on Col-Tau film (**A**). The high resolution XPS of C1s and N1s and split peak fitting analysis for Col (**B**,**D**) and Col-Tau (**C**,**E**).

Figure 1 shows the XPS results of the two samples. After chemical modification by the taurine, the sulfur element appears on the surface of Col-Tau (Figure 1A). Comparing the Col and Col-Tau film, the oxygen’s content increased from 18.87% to 19.83%, while the nitrogen’s content decreased from 13.85% to 12.11% by introducing taurine molecular (Table 1). The increase of oxygen and nitrogen content on the surface of the sample indicates that taurine molecular is successfully grafted on Col-Tau film by chemical conjugation. What’s more, the high resolution XPS curve of C 1s and N 1s is also presented in Figure 1. The high-resolution C 1s curve of the Col and Col-Tau that indicated a peak at about 285 eV can be resolved into four component peaks (Figure 1B,C). The peaks around 284.23 eV, 285.26 eV, 285.80 eV and 287.43 eV corresponded to the functional group C-C, C-N, C-O, and C=O, respectively. The high-resolution N 1s curve of Col and Col-Tau displayed the peak at about 400 eV, which can be fitted to two peaks: one at 399.45 eV and the other at 401.20 eV, are respectively corresponded to amine (-C-NH2) and amide (-CONH-) (Figure 1D,E).

### 3.2. Infrared Spectroscopy

**Figure 2:**
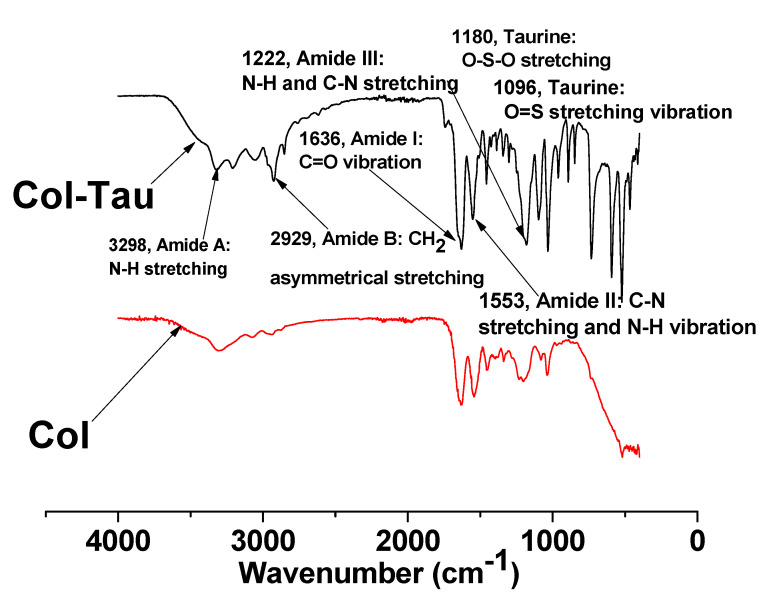
FT-IR of the Col and Col-Tau film within the wave number range of 4000–350 cm^−1^.

The Infrared spectroscopic analysis of two samples is shown in Figure 2. The test curve displayed that the new chemical bonds are formed between collagen and taurine molecular. As we predicted, the representative characteristic peaks of collagen are observed. The five peaks are corresponding to the amide I (1636 cm^−1^), amide II (1553 cm^−1^), amide III (1222 cm^−1^), amide A (3298 cm^−1^) and amide B (2929 cm^−1^) of the collagen molecule. The differences between the spectrums for two collagen-based samples are also appeared. Specifically, by moving from Col sample toward the Col-Tau sample, the spectrum shift upward toward higher absorption intensities. In addition, some characteristic peaks corresponding to the functional groups of taurine appears in the curve. FTIR spectra of Col-Tau depict taurine characteristic bands at 1180 cm^−1^ and 1096 cm^−1^ that are respectively attributed to O-S-O stretching absorption and O=S stretching vibration. Through the combination outcomes of infrared spectroscopy and XPS test, we believe that the taurine molecules were successfully grafted onto the surface of the Col-Tau film. 

### 3.3. Evaluation of Surface Contact Angle 

**Figure 3:**
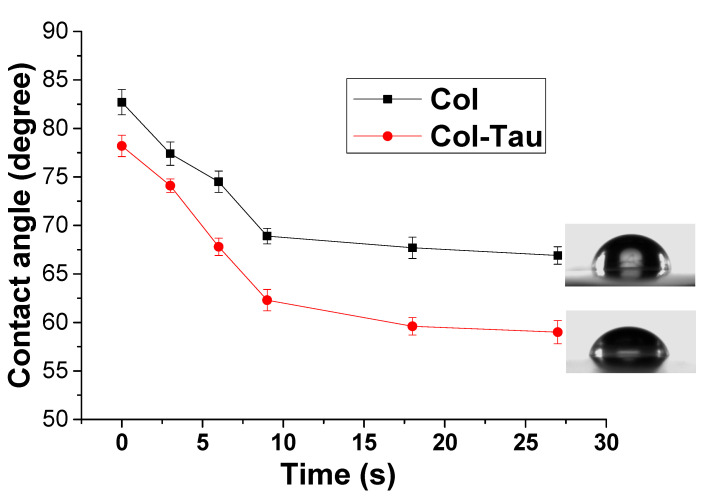
Variation trend of CA with time on the surface of Col and Col-Tau (n = 5). The CA of the collagen films decreased after the surface modification by taurine molecular.

The surface contact angle (CA) on the two films is shown in Figure 3. Water droplets spread out on the surface of the Col within 30 s and the CA decreased from 82.7 ± 1.3° to 66.9 ± 0.9°. Similarly, the CA on the Col-Tau film decreased from 78.2 ± 1.1° to 59.0 ± 1.2°. As shown in Figure 3, The CA of the collagen films decreased after the surface modification by taurine molecular. Therefore, we conclude that the surface of Col-Tau is more hydrophilic than that of the Col film. As mentioned in many studies [31], taurine has a hydrophilic molecular structure with amine and sulfonic groups, which leads to a higher polarity surface of the Col-Tau film than that of the blank Col sample, so the taurine-modified collagen sample exhibited a relatively minor CA. 

### 3.4. Saturated Water Absorption and Swelling Properties

**Figure 4:**
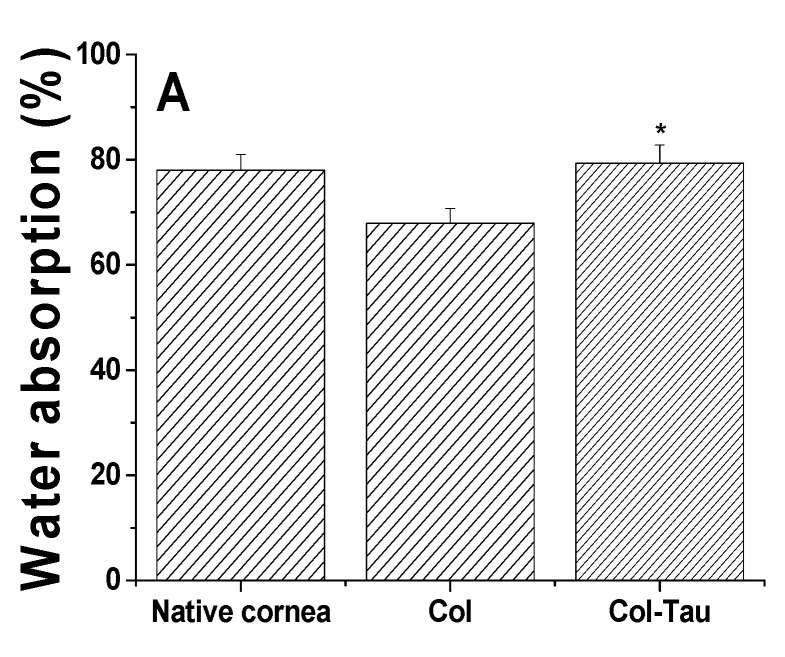

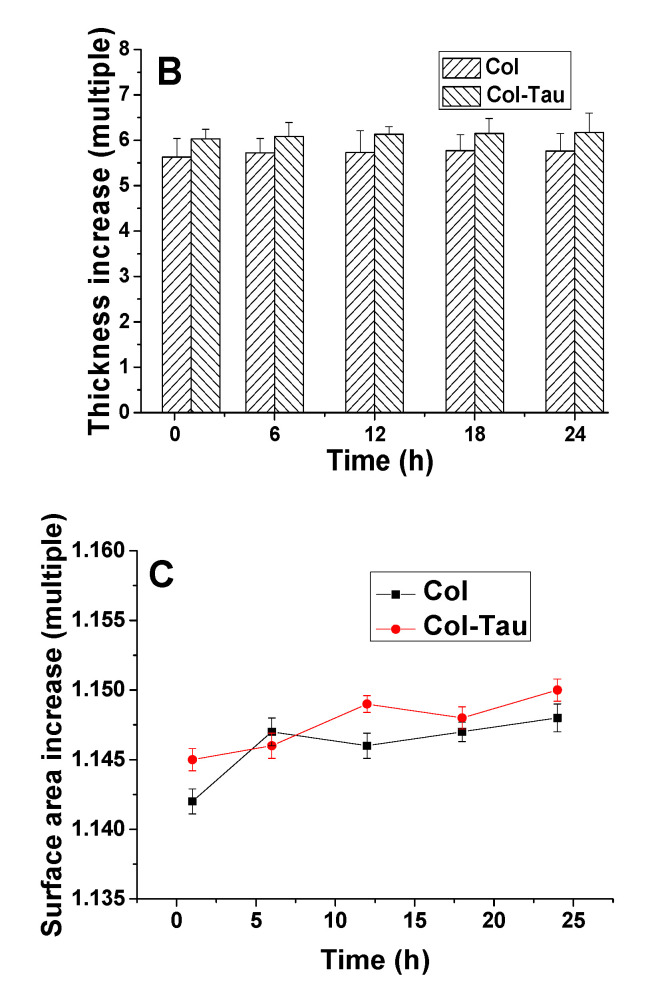
Water absorption performance of Col (control group), Col-Tau and native cornea (**A**). During the swelling of the films, variation of the two films’ thickness (**B**) and surface area (**C**) versus time. The original thickness of the dry films is about 25 ± 5 μm, and the original surface area of the dry films was 1 cm × 1 cm (n = 10). * significant differences between the experimental groups and the Col group.

As we know, moisture-retaining capacity plays the key role in maintaining the normal physiological function and structural stability of natural corneal [13]. Figure 4A exhibits the saturated water absorption of the two collagen-based samples. The water absorption performance of the Col-Tau film is 79.3 ± 3.5%. The results are very similar to the native cornea tissue (78.0 ± 3.0%), but significantly higher than the blank Col sample (67.9 ± 2.8%) [12,31]. The results indicate that the saturated water absorption of the Col-Tau film is promoted by the chemical grafting of the taurine’s sulfonic groups. 

Clinically, the extent and location of the corneal defects are different for each patient, so artificial grafts for corneal replacement should be easily fabricated with suitable size and thickness for corneal blind patients to choose from. Figure 4B exhibits the variation of the two collagen-based samples’ thickness versus time. The Col samples’ thickness increased 550% after water absorption and the change of Col-Tau’s thickness is higher than Col. Besides, Figure 4C shows the variation of the collagen-based samples’ surface size versus time. Both the two samples maintained a relatively stable size after saturated water absorption. This correlates to the fact that the two films have similar dense structures. The change in the Col’s surface size was slightly lower than Col-Tau. This may be related to the introduction of taurine molecules. According to the above results, the dry and wet dimensions of the two collagen-based films are repeatable and controllable. Therefore, we think this Col-Tau film can be made with different morphology easily and has application potential for personalized corneal replacement. 

### 3.5. Transparency of the Collagen-Based Films

**Figure 5:**
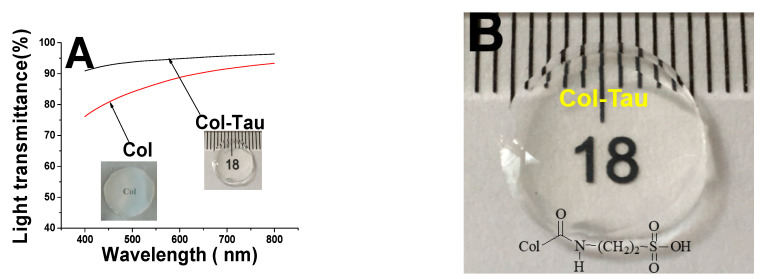
Optical performance of the Col-Tau and Col film (**A**). The pre-wetted Col-Tau film is almost transparent (**B**).

The optical performance of the two collagen-based samples is shown in Figure 5A. Both two films exhibited good transparency. The light transmittance of the pre-wetted Col-Tau and Col sample indicate minor differences. With the increase of wavelength, the light transmittance curve of the collagen-based films reached the highest level (higher than 90%), which is very similar to that of natural cornea tissue (about 87%) [11,12,31]. In addition, Figure 5B exhibits a picture of pre-wetted Col-Tau sample. The introduction of taurine has little effect on the optical performance of the Col film.

### 3.6. Mechanical Performance

**Figure 6:**
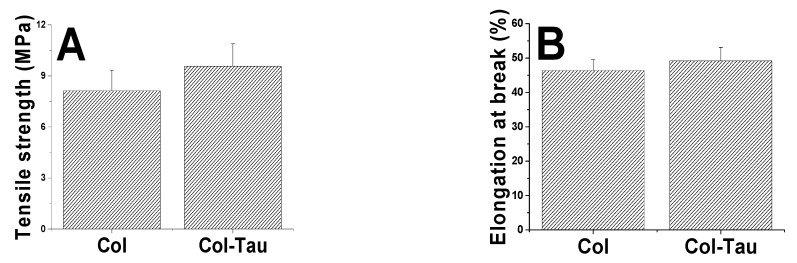

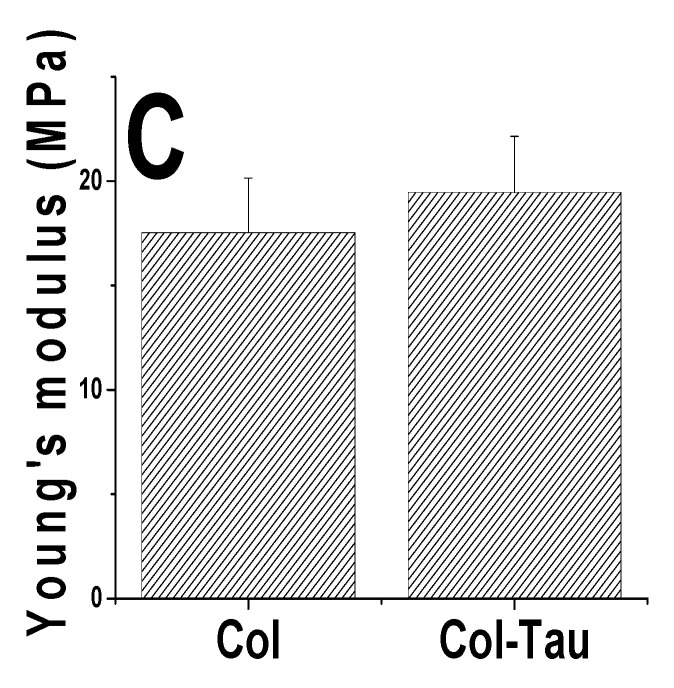
Mechanical performances of the hydrated Col and Col-Tau film. The three representative indicators are as follows: tensile strength (**A**), elongation at break (**B**) and the Young’s modulus (**C**). Values are expressed as the mean ± standard deviation (n = 5).

Before testings, the samples were rehydrated until they reached saturation. The tensile strength of hydrated Col-Tau and Col was 9.57 ± 1.32 MPa and 8.13 ± 1.21MPa (Figure 6A), respectively. At the same time, the elongation at break has little difference between Col-Tau (49.17 ± 3.92%) and Col (46.37 ± 3.21%) (Figure 6B). Furthermore, according to the about results, we calculated that Young’s modulus of the hydrated Col-Tau and Col was 19.46 ± 2.69 MPa and 17.53 ± 2.61 MPa (Figure 6C), respectively. Compared with the pure collagen film, the hydrated Col-Tau film exhibited suitable mechanical properties. Moreover, the tensile strength of Col-Tau film was similar as human corneal tissue (about 11 MPa) [20,31]. Apart from the moisture-retaining capacity, taurine also plays another role. In the part close to the surface layer of the collagen film, after chemical modification with taurine, the intermolecular forces within the collagen-based material are enhanced, and the movement or migration of these macromolecules will also require more energy. For that reason, the mechanical properties of Col-Tau film were slightly improved macroscopically.

### 3.7. The Metabolic Activity of PC12 Neuronal Cells on Col and Col-Tau

**Figure 7:**
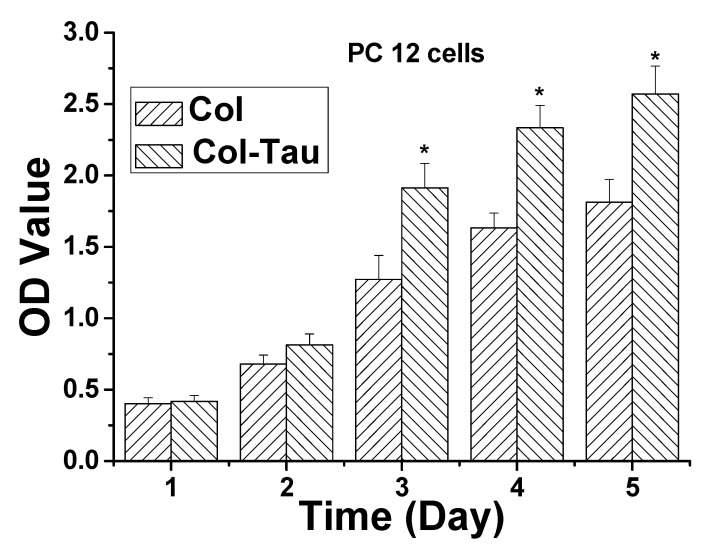
The metabolic activity of PC12 neuronal cells on Col-Tau and Col (blank group) film (MTT measured at 490 nm). The growth rate of PC12 cells on the Col-Tau film was significantly faster than on the Col film, which confirms that the introduction of taurine can effectively promote cell growth of PC 12 cells. * significant differences between the experimental groups and the Col group.

PC12 cells have been used as a good model system for neuronal growth and differentiation in many studies [29,30]. The metabolic activity of PC12 cells on the Col-Tau and Col film within one week was quantificationally evaluated by the MTT test. Figure 7 indicates the proliferation rate of PC12 cells on the two kinds of collagen-based film. After the PC12 cells were culture with samples, the cells had similar growth rates on the two kinds of films on day 1. However, the PC12 cell’s growth rate on the Col is lower than that of the Col-Tau during the subsequent time. We think that the introduction of taurine molecules on the surface of Col-Tau allows it combines with the proteins in the cell culture medium easily [28]. Therefore, the Col-Tau’s surface is more conducive to binding cell-adhesive proteins and subsequent cell adhesion, thereby accelerating the proliferation process of PC12 cells. The MTT results confirm that the introduction of taurine can effectively promote cell adhesion and the proliferation rate of PC12 cells.

### 3.8. The HCECs Growth and Epithelization Process on Col-Tau Film

**Figure 8:**
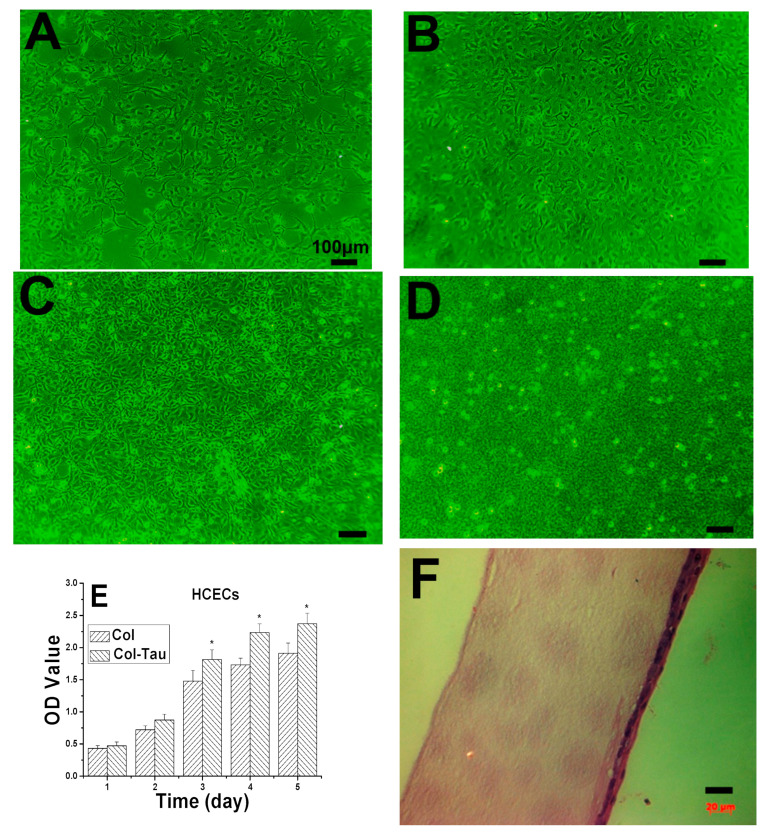
The change in HCECs’ morphology on Col-Tau film at target time points: 0.5 day (**A**), one day (**B**), two days (**C**) and three days (**D**). The proliferation rate of HCECs on the Col-Tau and Col film (**E**) (n = 10). The * represents a significant difference between the Col and Col-Tau. After the cells grow on the surface of the Col-Tau film for one week. The photo of the H&E staining section exhibits that about 2–3 layers of cells covered the surface of the Col-Tau (scale bar = 20 μm) (**F**).

Because the cornea is a sensitive and highly protected organ, cell proliferation and adhesion are essential in determining the availability of artificial material for corneal replacement [13,16]. Human corneal epithelial cells (HCECs) were used to study the cytocompatibility of the two collagen-based films. Figure 8 exhibits the change of the HCECs’ morphology after being cultured on Col-Tau film at target time points. After the cells were incubated for 0.5 day (Figure 8A), one day (Figure 8B), two days (Figure 8C) and three days (Figure 8D), the HCECs attached and exhibited a widespread on the Col-Tau film. As seen in Figure 8A, the seeded HCECs adhered to the surface of the samples quite well within the 12 h. The results indicated that Col-Tau film has relatively good cell adhesion, which is consistent with the experiment results of cell proliferation. What’s more, it is noteworthy that the cellular morphology of HCECs on the Col-Tau film’s surface was gradually changed from a spherical shape to the native cornea’s spindle shape. 

The metabolic activity of HCECs on the Col-Tau and Col at 1–5 consecutive days were quantitatively evaluated by MTT test. Figure 8E shows the process of cell proliferation on the Col-Tau and Col film, respectively. When the HCECs were transferred to the Petri dishes that containing the films, the cells proliferated rapidly. We found that the Col-Tau film was almost completely covered by the HCECs after incubation for about 72 h. In fact, the growth rate of HCECs is almost the same in the first 12 h. Then, same as the PC12 cells, the epithelial cell’s growth rates on Col-Tau film are significantly higher than that on the Col in the next few days. The results of cell experiments demonstrated that the introduction of taurine accelerated the epithelialization process of corneal epithelial cells on the collagen-based material. What’s more, Figure 8F is the H&E staining photo of the cross-section of Col-Tau, which had been used for HCECs culture for seven days. The picture of histological sections exhibits that about 2–3 layers of cells had covered the whole surface of the Col-Tau. Combined with the above cell adhesion and proliferation experiments, these results show that the HCECs can be well combined onto the surface of Col-Tau. All the results of cell experiments demonstrated the excellent cytocompatibility of this Col-Tau film.

## 4. Conclusions

In this work, we proposed new collagen-taurine film with good biocompatibility and biological functionality for cornea repair. The results indicate that this new collagen-based material has a similar mechanical and optical performance to the human cornea. Besides, this Col-Tau film also reproduced the key features of the native tissue, such as high hydrophilicity and moisture retention capacity, which can decrease the xerophthalmia risk after corneal transplantation in a clinic. Furthermore, the cell experiment results show excellent capacity of this functionalized collagen film to promote corneal nerve repair and the epithelization process. This Col-Tau material, which can be prepared easily, should have significant application potential for visual rehabilitation in the future.

## Figures and Tables

**Table 1:** After chemical modification, the surface chemical compositions of Col and Col-Tau film.

Sample	C (%)	O (%)	N (%)	S (%)	N/C	O/C
**Col**	67.28	18.87	13.85	0	0.21	0.28
**Col-Tau**	67.27	19.83	12.11	0.79	0.18	0.29

## Data Availability

Data are included in the text; raw data are available from the corresponding authors.
